# Constitutive ß-Catenin Signaling by the Viral Chemokine Receptor US28

**DOI:** 10.1371/journal.pone.0048935

**Published:** 2012-11-08

**Authors:** Ellen V. Langemeijer, Erik Slinger, Sabrina de Munnik, Andreas Schreiber, David Maussang, Henry Vischer, Folkert Verkaar, Rob Leurs, Marco Siderius, Martine J. Smit

**Affiliations:** Division of Medicinal Chemistry, Leiden/Amsterdam Center for Drug Research, VU University Amsterdam, The Netherlands; Northwestern University Feinberg School of Medicine, United States of America

## Abstract

Chronic activation of Wnt/ß-catenin signaling is found in a variety of human malignancies including melanoma, colorectal and hepatocellular carcinomas. Interestingly, expression of the HCMV-encoded chemokine receptor US28 in intestinal epithelial cells promotes intestinal neoplasia in transgenic mice, which is associated with increased nuclear accumulation of ß-catenin. In this study we show that this viral receptor constitutively activates ß-catenin and enhances ß-catenin-dependent transcription. Our data illustrate that this viral receptor does not activate ß-catenin via the classical Wnt/Frizzled signaling pathway. Analysis of US28 mediated signaling indicates the involvement of the Rho-Rho kinase (ROCK) pathway in the activation of ß-catenin. Moreover, cells infected with HCMV show significant increases in ß-catenin stabilization and signaling, which is mediated to a large extent by expression of US28. The modulation of the ß-catenin signal transduction pathway by a viral chemokine receptor provides alternative regulation of this pathway, with potential relevance for the development of colon cancer and virus-associated diseases.

## Introduction

The Wnt/ß-catenin signaling pathway plays critical roles in embryonic development, stem cell self-renewal and regeneration [1], [2]. Perturbations in this signaling cascade have been implicated in the pathogenesis of cancer. Notably, chronic activation of Wnt/ß-catenin signaling is found in a variety of human malignancies including melanoma, colorectal and hepatocellular carcinomas [3], [4]. Accordingly, components of the Wnt/ß-catenin pathway are important targets for cancer therapeutics [3]. In the absence of an extracellular Wnt ligand, cytoplasmic ß-catenin is phosphorylated through the action of the “destruction complex”, a large protein assembly that contains the Ser/Thr kinases casein kinase 1α (CK1), glycogen synthase kinase 3 (GSK-3) and the tumor suppressors Axin and Adenomatous polyposis coli (APC) [1]. Phosphorylation of ß-catenin targets it for ubiquitin-mediated proteasomal degradation. However, upon stimulation of the seven-transmembrane receptor Frizzled and the single-pass low-density lipoprotein receptor-related protein LRP5/6 by a Wnt ligand, the destruction complex function is compromised through a not fully understood mechanism. As a result, ß-catenin will not be phosphorylated and will no longer be subject to degradation, and will subsequently translocate to the nucleus [5]. Nuclear ß-catenin functions as a transcriptional co-activator of target genes such as c*-myc* and cyclin D1, which are involved in proliferation, survival and oncogenic transformation [6], [7], [8].

The importance of GPCR-mediated signaling in onset and development of various types cancer [9] is underscored by the observation that ß-catenin activation is triggered by a 7TM spanning receptor, Frizzled which is activated by its cognate ligand Wnt [1]. Besides Frizzled receptors, a few other G protein-coupled receptors (GPCRs) mediate ß-catenin induced transcriptional activation [10], [11]. The lysophosphatidic acid LPA2 receptor and LPA3 both trigger ß-catenin stabilization and cell proliferation via protein kinase C activation [12]. Additionally, the pro-inflammatory metabolite prostaglandin E2 activates ß-catenin through activation of its cognate receptor [13]. The human protease-activated receptor-1 (PAR-1) stabilizes ß-catenin through phosphorylation of GSK-3ß at Ser9. Altogether, these pathways converge on the Wnt signaling route to induce cytoplasmic ß-catenin accumulation, nuclear localization, and enhanced transcriptional activation [14].

In this study we show that the human cytomegalovirus (HCMV)-encoded GPCR US28 positively modulates ß-catenin signaling, resulting in enhanced ß-catenin-dependent transcription. US28 is one of four GPCRs encoded by the HCMV [15]. Interestingly, this widely spread ß-herpesvirus [16] has been associated with vascular diseases [17] and has been suggested to act as an oncomodulator [18]. All four HCMV-encoded GPCRs (vGPCRs) show high homology to human chemokine receptors, which play a fundamental role in the control and regulation of the immune system and in the progression of cancer and metastasis [19], [20]. US28 is able to signal in a constitutive, ligand-independent, manner via Gα_q_ and Gßγ but also in a ligand-dependent manner via Gα_12_ to proliferative and pro-angiogenic signaling pathways [15], [21], [22]. US28 has oncogenic properties as US28-expressing NIH-3T3 cells promote tumorigenesis when injected into nude mice [27]. Moreover, US28 expression was detected in human glioblastomas and medulloblastomas, which was associated with increased STAT3/IL-6 and COX-2 activity [23], [24], [25] Moreover, transgenic mice expressing US28 in intestinal epithelial cells, including LGR5-positive stem cells, develop adenomas and adenocarcinomas, associated with increases in ß-catenin protein stabilization and nuclear localization [26]. Additionally, transcriptional profiling of US28-expressing fibroblasts indicated also an overrepresentation of genes involved in the Wnt/ß-catenin signaling pathway [27]. These observations suggest that US28 may facilitate transformation and development of intestinal neoplasia via activation of ß-catenin [26].

**Figure 1:**
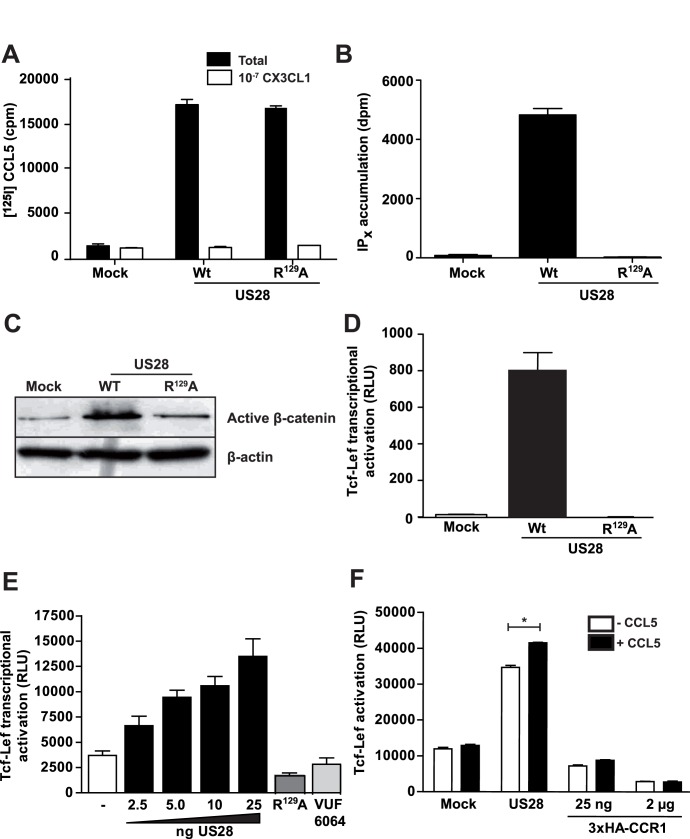
US28 induces constitutively activates ß-catenin signaling. US28 is expressed and functional in NIH-3T3 cells. A, Whole cell binding of [^125^I]-CCL5 on NIH-3T3 cells expressing wild-type (WT), mutant R^129^A or HA-tagged US28 is displaced by fractalkine (CX3CL1). B, US28-WT constitutively stimulates inositol phosphate (IPx) accumulation in NIH-3T3, while the non-G protein-coupling mutant US28-R^129^A shows no activation. C, Total cell extracts of NIH-3T3 cells stably expressing US28, the non G-protein-coupling mutant and empty plasmid control (mock) were analysed on Western blot with antibodies recognizing the non-phospho (active) ß-catenin, total ß-catenin and actin. D, NIH-3T3 cells stably expressing US28 and the non G-protein coupling US28 mutant R^129^A were transfected with the Tcf-Lef reportergene construct. Luciferase activity was measured 24 h after transfection. E, US28 dose-dependently induces Tcf-Lef transcriptional activation in HEK293T cells. The non-G protein-coupling mutant US28 R129A does not display activation of the reportergene at 25 ng DNA transfected (dark grey bar). Treatment of HEK293T cells transfected with 25 ng US28 DNA with inverse agonist VUF 6064 (10 µM) prevents activation of Tcf-Lef reortergene (light grey bar). F, HEK293T cells transfected with the human chemokine receptor CCR1 and the Tcf-Lef reportergene construct do not show Tcf-Lef activation nor after exposure to 100 nM CCL5 (RANTES). US28 expressing HEK293T cells display constitutive signaling to the Tcf-Lef reportergene, which is significantly enhanced by exposure to 100 nM CCL5 (RANTES).

In this study we show that the viral chemokine receptor US28 positively modulates the ß-catenin pathway via a non-conventional novel pathway, involving Rho kinase.

## Experimental Procedures

### Cell Culture and Transfections

Human HEK293T, human glioblastoma U373-MG, human foreskin fibroblasts (HFF) and NIH-3T3 mouse fibroblast cells were all obtained from ATCC, and cultured in Dulbecco’s Modified Eagle’s Medium (DMEM) (PAA Laboratories), supplemented with penicillin (50 IU/ml), streptomycin (50 µg/ml) (PAA Laboratories) and 10% fetal bovine serum (FBS) (PAA Laboratories), heat-inactivated FBS and bovine serum (Gibco) respectively. NIH-3T3-stable cell lines (Mock, US28, HA-US28 and US28-R^129^A mutant) were kept under the selective pressure of neomycin (400 µg/ml) (Sigma) to ensure homogenous receptor expression. Transient transfections of HEK293T cells were performed with the polyelthyleneimine (PEI) method [28], [29] followed by luciferase activity measurement the next day. Transient transfections of NIH-3T3 and U373-MG cells were performed with the Lipofectamine2000 method (Invitrogen).

**Figure 2:**
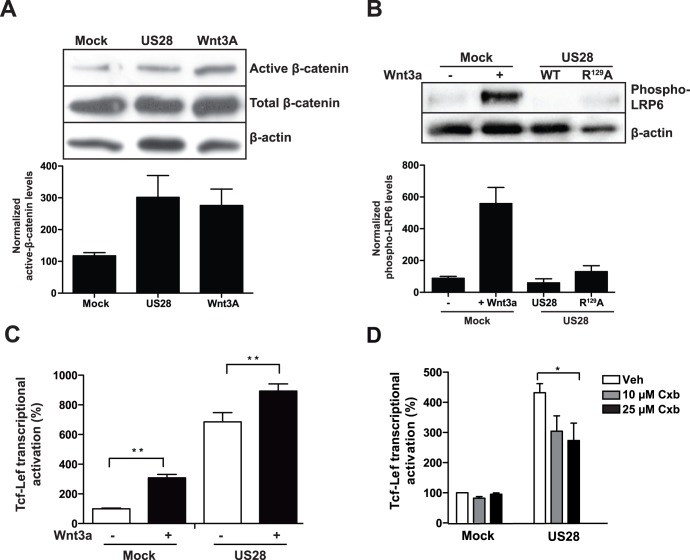
Classical Wnt/Frizzled/ß-catenin **signaling is not involved in US28-mediated Tcf-Lef activation.** A, Western blot analysis of total cell extracts of NIH-3T3 cells, stably expressing US28 or an empty plasmid (mock) which were treated with Wnt3a- (overnight, 200 ng/ml) and vehicle-treated mock cells. The blot was probed with antibodies recognizing the non-phosphorylated (active ß-catenin, total ß-catenin and actin. A representative blot is shown and normalized quantifications of (active) ß-catenin of independent experiments are shown below the blot. B, Western blot analysis of total cell extracts of NIH-3T3 cells stably expressing US28, the non G-protein coupling US28 mutant R^129^A or an empty plasmid (mock) and Wnt3a-treated mock cells. The blot was probed with antibodies recognizing Lrp6-phospho-ser^1490^ and actin. A representative blot is shown and normalized quantifications of Lrp6-phospho-ser^1490^ of independent experiments are shown below the blot. C, HEK293T cells co-transfected with the Tcf-Lef reporter gene construct and either US28-expressing or an empty control plasmid (mock) exposed to Wnt3a (overnight, 200 ng/ml). Luciferase activity was measured 24 hr after transfection and is displayed here as the percentage of the non-treated mock control that is set at 100%. D, HEK293T cells co- transfected with the Tcf-Lef reportergene and an US28-expressing construct or empty plasmid control (mock) were exposed to various concentrations (ON, 10–25 µM) of the COX2 inhibitor celecoxib (Cxb). Tcf-Lef reporter gene activation was measured 24 hr after transfection and is displayed here as the percentage of the mock control that is set at 100%.

### Reporter Gene Analysis

10^6^ HEK293T cells were transfected with plasmids encoding a TOP-flash reporter construct (TOPflash or the negative control FOPflash, kindly provided by Prof. H. Clevers and Dr. M. vd Wetering) and 25 ng of US28 receptor DNA (wild type or G-protein-uncoupled mutant R^129^A) unless indicated differently and 25 ng of DNA encoding G-proteins (RGS2 and Lsc-RGS G protein scavengers were kindly provided by Dr. B. Moepps). Comparable TOPflash reportergene transfection protocols were used for U373-MG and NIH-3T3 cells, respectively. Total DNA amounts were kept constant by addition of empty vector. Inhibitors Y27632 (Rock, Sigma) were incubated overnight and added directly after transfection. 200 ng/ml human recombinant Wnt3a (R&D systems, 5036-WN-010) was incubated overnight to activate the canonical Wnt signaling pathway. Luciferase activity was measured 24 h post transfection (RLU, relative light units) with a Victor^2^ multilabel plate reader from (PerkinElmer Life Sciences). Statistical analyses, * or ** indicating p<0.05 or p<0.001, using Anova and Bonferroni post test 95% confidence interval.

**Figure 3:**
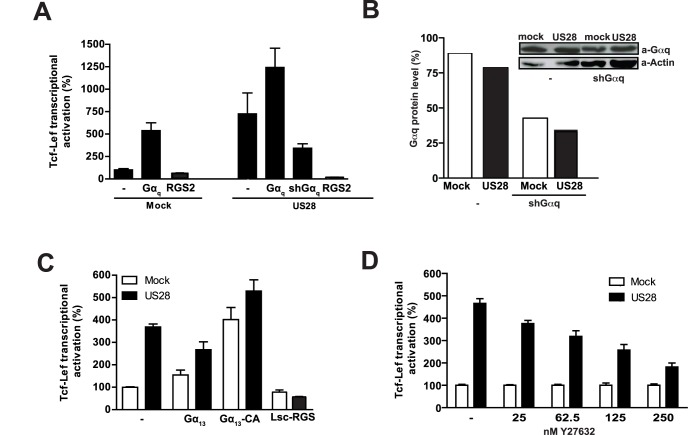
G protein involvement in US28-mediated Tcf-Lef activation. A, HEK293T cells were co-transfected with the Tcf-Lef reporter gene construct, a US28-expressing construct or empty plasmid control (mock) and various constructs expressing Gα-proteins as indicated, Gα_q-11_ shRNA construct or a construct expressing regulator of G protein signaling 2 (RGS2), known to specifically interfere with Gα_q_ signaling. Tcf-Lef reporter gene activation was measured 24 hr after transfection and is displayed here as the percentage of the mock control that is set at 100%. B, HEK293T cells were co-transfected with the Tcf-Lef reporter gene construct, US28-expressing construct or empty plasmid control (mock) and an shRNA construct to decrease protein levels of Gα_q_. Total cell extracts were analysed on Western blot using antibodies recognizing Gα_q_ or actin (insert). Bars represent level of Gα_q_ protein level compared to the actin levels, with the ratio in non-treated mock cells set at 100%. C, HEK293T cells were co-transfected with the Tcf-Lef reporter gene construct, a US28-expressing construct or empty plasmid control (mock) and various constructs expressing Gα_13_, a constitutive active (CA) Gα_13_ or Lsc-RGS, encoding the RGS domain of the Rho GTPase guanine nucleotide exchange factor (Rho-GEF) Lsc, known to specifically interfere with transmembrane signaling mediated by activated Gα_12/13_. Tcf-Lef reporter gene activation was measured 24 hr after transfection and is displayed here as the percentage of the mock control that is set at 100%. D, HEK293T cells co-transfected with the Tcf-Lef reporter gene construct, a US28-expressing construct or empty plasmid control (mock) were treated (overnight) with various concentrations of the ROCK inhibitor Y27632 as indicated.

### Virus Infection

Human Foreskin Fibroblasts (HFF) infected at a multiplicity of infection (MOI) of 1 on IBIDI slides with the TB40wt and TB40-ΔUS28 strains, respectively. Anti-IEA (Milipore) and anti-non-phospho-ß-catenin antibodies (Cell Signaling Technology) were used to visualize IEA and activated ß-catenin. After transfection (24 h) of the TOPflash reporter gene in U373-MG cells different HCMV Titan strains (WT or -ΔUS28) were used to infect U373-MG at an MOI of 2. Multiple viral stocks (3 for HCMV-WT, 4 for -ΔUS28) were assayed in triplicate. The rate of infectivity was controlled by back titration and IEA staining on parallel clear plates. 48 h post-infection luciferase activity was measured.

**Figure 4:**
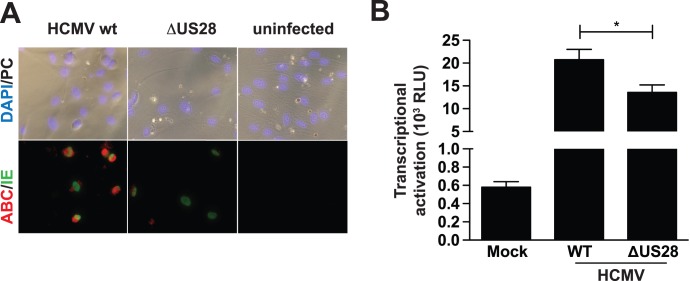
HCMV-infected cells stimulate activation of ß-catenin in a US28 dependent manner. A. HFF cells were infected with HCMV-WT or HCMV-ΔUS28 with a M.O.I of 1 on IBIDI slides. Cells were fixed 24 hours post-infection (hpi) and stained with antibodies recognizing the HCMV immediate early antigen (IEA) and activated ß-catenin respectively. B. U373-MG cells transfected with Tcf-Lef reporter gene were either infected with HCMV-WT or HCMV-ΔUS28 with a M.O.I. of 2, or left uninfected (mock). Luciferase activity was measured 48 h post-infection.

### Chemokine Binding and Inositol Phosphate Accumulation Experiments

Stably transfected NIH-3T3 cells (Mock, HA-US28 and US28) were analyzed for radiolabelled chemokine binding and inositol phosphate accumulation as previously described [15].

**Figure 5:**
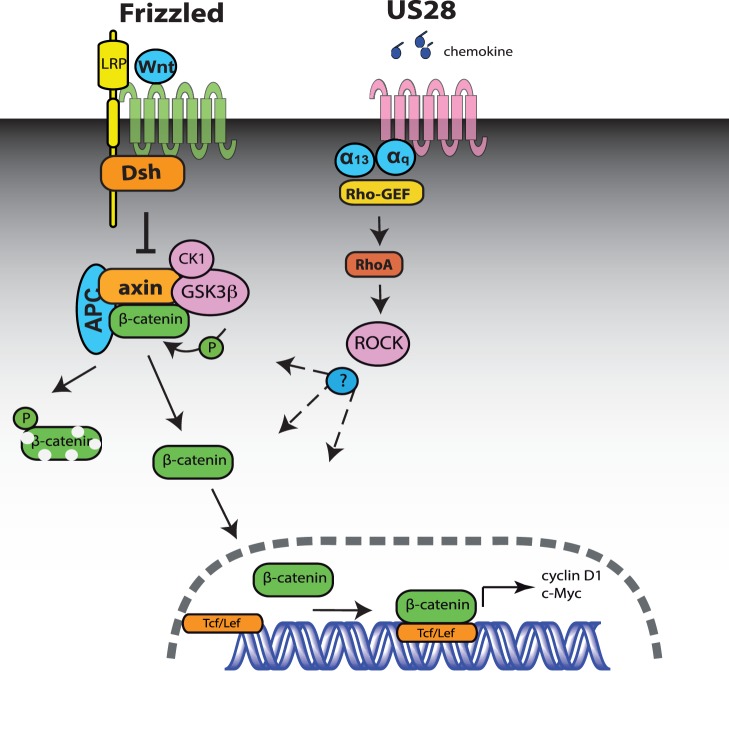
Schematic representation of the classic Wnt signaling pathway and model of US28-mediated activation of ß-catenin signaling pathway. The left side of the model indicates components of the classic Wnt/Frizzled mediated activation of ß-catenin. In this pathway the disruption complex (Axin, APC) that enables GSK3ß- and Caseine kinase 1 (CK1)-mediated phosphorylation of ß-catenin leading to its degradation, is disrupted in a Dishevelled-mediated way upon Wnt binding to Frizzled/LRP. US28 activates ß-catenin signaling in a ligand-dependent and independent manner, involving respectively Gα_12/13_ and Gα_q_ proteins and respective RhoGEFs, converging at RhoA/ROCK, resulting in increased Tcf-Lef transcriptional activation.

### Western Blot Analysis

Biorad minigel and electroblot systems (Biorad) were used to perform SDS-PAGE and subsequent protein transfer onto 0.45 µm nitrocellulose or PVDF membranes. After an overnight serum starvation in medium containing 0.5% bovine serum, NIH-3T3 stable cell lines (Mock, US28 and US28-R^129^A) were lysed in radioimmunoprecipitation assay buffer supplemented with α-Complete Protease Inhibitor Cocktail (Hoffmann-la Roche), 1 mM PMSF, 1 mM NaVO_4_ and 1 mM NaF. Samples were normalized using the BCA total protein determination kit (Thermo Fisher Scientific, Rockford lL, USA). Antibodies were used for detection of active ß-catenin (Millipore and Cell Signaling Technology), total ß-catenin (BD Transduction Laboratories), mouse monoclonal ß-actin expression (Sigma), Gα_q_ (Santa-Cruz) and P-LRP6 (Ser^1490^) (Cell Signaling Technology).

## Results

### Viral Chemokine Receptor US28 Activates the ß-catenin Pathway

Transgenic mice expressing US28 in intestinal epithelial cells develop adenomas and adenocarcinomas that express high levels of nuclear ß-catenin protein [26]. Additionally, US28-mediated up-regulation of genes associated with ß-catenin signaling was described [27]. These findings and the role of ß-catenin in oncogenesis [1], [2], prompted us to investigate the mechanism by which this viral GPCR activates ß-catenin signaling. Since this receptor displays constitutive active properties [30] we used the wild type (WT) as well as a G protein-uncoupled mutant receptor (US28-R^129^A) in these studies. US28-WT expressing NIH-3T3 cells displayed [^125^I]-CCL5 binding and increased inositol phosphate production compared to mock-transfected cells (Fig. 1A,B), indicative of proper plasma membrane targeting and functionality of the receptor. Cells expressing the G protein-uncoupled mutant US28 (US28-R^129^A) receptor displayed [^125^I]-CCL5 binding but no constitutive signaling (**Fig. 1A, B**). Using Western blot analysis, non-phospho (active) ß-catenin levels were shown to be elevated in US28-expressing NIH-3T3 cells compared to mock-transfected and US28-R^129^A expressing cells (**Fig. 1C**). Further indication for US28-mediated activation of ß-catenin signaling was generated using a ß-catenin -specific reporter gene construct containing TCF/Lef binding sites (TOPflash) [31]. Analysis of activation of TOPflash in the NIH-3T3 cells stably expressing US28 confirmed US28-mediated activation of ß-catenin signaling (**Fig. 1D**). As expected, stable expression of the G protein-uncoupled mutant (US28-R^129^A) did not display ß-catenin activation. Also in HEK293T cells, increasing expression of US28 resulted in dose-dependent activation of the ß-catenin signaling pathway as demonstrated using the TOPflash reporter gene (**Fig. 1E**). A reporter gene containing mutant TCF/Lef-binding sites (FOPflash), used as a negative control, was not induced in US28 expressing cells (data not shown). In accordance with the Western blot data, cells expressing the G protein-uncoupled mutant US28-R^129^A did not display TOPflash reporter gene activity, indicating the importance of G-protein signaling in US28-induced activation of the ß-catenin signaling pathway (**Fig. 1E**). Treatment of US28 transfected cells with the US28 small molecule inverse agonist VUF6064 [32] at a 10 µM concentration, prevented activation of the Tcf-Lef reporter gene construct (**Fig. 1E**).

To investigate whether US28-mediated ß-catenin signaling can be modulated by chemokine ligands, we stimulated US28-expressing cells with CCL5, which enhances US28-dependent signaling through Gα_q_ and Gα_12_ pathways [33]. Stimulation of US28 with 100 nM CCL5 yielded a small but significant increase in TOPflash reporter gene activity (**Fig. 1F**). Expression of CCR1, a human chemokine receptor displaying close homology to US28, did not induce activation of TOPflash, neither in a constitutive manner, nor upon stimulation with 100 nM of its endogenous ligand CCL5 (**Fig. 1F**).

### US28 Activates ß-catenin/TOPflash via a Non-classical Signal Transduction Pathway

We next compared the mechanism by which the classical activator of the Wnt/Frizzled pathway Wnt3a and US28 activate ß-catenin signaling. As depicted in **Fig. 2A**, both Wnt3a [200 ng/ml] and US28 expression induced stabilization of ß-catenin in NIH3T3 cells. Activation of ß-catenin through the classical Wnt/Frizzled pathway involves the CK1γ/GSK3ß-mediated phosphorylation of LRP 5/6, which leads to recruitment of Axin and Dishevelled to the plasma membrane, culminating in the disruption of the destruction complex [34], [35]. Analysis of LRP6 phosphorylation in US28-expressing cells indicated that in contrast to Wnt3a-stimulated ß-catenin activation of either mock or US28 transfected cells, LRP6 phosphorylation was absent at serine residue 1490, suggesting an alternative mechanism of ß-catenin activation for US28 (**Fig. 2B** **and Fig. S1**). In line with this notion, activation of TOPflash activity through Wnt3a and US28 were additive (**Fig. 2C**), suggesting parallel modes of pathway activation. These data illustrate that US28 does not activate β-catenin via the classical Wnt/Frizzled signaling pathway. To investigate this alternative mechanism of US28-mediated ß-catenin signaling, various inhibitors of potential effectors of the GSK-3ß/APC destruction complex, such as PLC (U73122), PKC (203291), Akt (124005), PI3K (Wortmannin and LY294002), Src (PP-2) and STAT-3 (Stattic) were analysed as potential modulators of US28-mediated ß-catenin activation. Neither of these showed any effect on US28-induced TOPflash reporter gene activation (data not shown).

Earlier studies attributed a role for COX-2 in US28-mediated cellular responses [23], [27]. Since COX-2 activation has been linked to activation of the ß-catenin pathway [13] we analysed the effect of celecoxib (COX-2 inhibitor) on US28-mediated ß-catenin-dependent reporter gene activity. Celecoxib treatment inhibited the TOPflash reporter gene activity only partially at relatively high celecoxib concentrations, indicating only a minor contribution of COX-2 (**Fig. 2D**).

### G protein Involvement in US28 Enhanced TOPflash Reporter Gene Activation

The complete lack of activity of the G protein-uncoupled receptor US28-R^129^A mutant towards ß-catenin signaling suggested that G protein coupling is essential for activation of ß-catenin by US28. The involvement of G-proteins in US28-mediated TOPflash reporter gene activation was further examined by co-expressing different G protein subunits or by co-expressing constructs known to negatively regulate G protein function. Co-transfection of the Gα-proteins Gα_s_, Gα_i2_, Gα_i3_, Gα_11_, Gα_13_ with US28 did not influence TOPflash reporter gene activation nor did overnight treatment with the Gα_i_–specific inhibitor pertussis toxin (PTX) (data not shown). Co-expression of Gα_q_ enhanced US28-mediated TOPflash reporter gene activation, while knocking down Gα_q_ with shGα_q_ resulted in over 50% inhibition of US28-mediated TOPflash activation (**Fig. 3A**). Downregulation of Gα_q_ protein levels was confirmed by Western blot analysis (**Fig. 3B**). Expression of the regulator of G protein signaling 2 (RGS2), which is known to specifically interfere with Gα_q_-mediated signaling [36] strongly reduced US28-induced TOPflash activation (**Fig 3A**). Interestingly, co-transfection of a constitutively active mutant of Gα_13_ (Gα_13_-CA), but not wild type Gα_13,_ resulted in enhanced TOPflash activation in mock cells (**Fig. 3C**). This effect was enhanced when US28 was co-transfected. Finally, we co-transfected cells with US28 and the Lsc-RGS scavenger, encoding the RGS domain of the Rho GTPase guanine nucleotide exchange factor (Rho-GEF) Lsc. Lsc-RGS is known to specifically interfere with transmembrane signaling mediated by activated Gα_12/13_ signaling [36], [37]. Expression of Lsc-RGS in US28-expressing cells resulted in a strong inhibition of US28-mediated TOPflash reporter gene activation (**Fig. 3C**), indicating an important role for Gα_12/13_ proteins.

As Gα_q_ and Gα_12/13_ proteins mediate activation of Rho via the Rho-GEFs p63 and p115 [37], [38], respectively and Rho in turn is known to activate ROCK kinase we investigated the role of the Rho-ROCK pathway in US28-mediated signaling to ß-catenin. Exposure of US28-expressing cells to increasing concentrations of the ROCK inhibitor Y27632 resulted in a dose-dependent attenuation of the US28 mediated TOPflash activation (**Fig. 3D**), indicating involvement of Rho-ROCK signaling in the US28-induced ß-catenin activation pathway.

### Role for US28 in HCMV Induced Activation of ß-catenin Signaling

The HCMV Titan 2B strain (referred to as WT) [27] and a strain deficient for the US28 gene (HCMV-ΔUS28) were used to examine the ability of HCMV, and the possible contribution of US28, in activating ß-catenin signaling after infection. Infection of human foreskin fibroblasts (HFFs) with HCMV-WT resulted in increased presence of active ß-catenin in cytoplasm and nuclei of infected cells, as evidenced by the expression of the immediate early antigen (IEA) (**Fig. 4A**). Cells infected with the deletion mutant HCMV-ΔUS28 showed only marginal active ß-catenin in these cells and no active ß-catenin was apparent in non-infected cells (**Fig. 4A**). Since HFFs show low transfection efficiencies we used U373-MG glioma cells to transfect the TOPflash reporter gene construct to monitor ß-catenin-dependent transcriptional activation after HCMV infection. U373-MG glioma cells were transfected with the TOPflash reporter gene construct, followed by infection with either HCMV-WT or HCMV-ΔUS28. Cells infected with HCMV-WT showed strong ß-catenin-dependent transcriptional activation (**Fig. 4B**), while cells infected with the deletion mutant HCMV-ΔUS28 displayed a significantly lower level of TOPflash reporter gene activity (**Fig. 4B**). The levels of infection between HCMV-WT and HCMV-ΔUS28 were similar, as determined by back titration (**Fig. S2**). These data clearly indicate a role for US28 in regulation of ß-catenin signaling during HMCV-infection.

## Discussion

We have demonstrated that HCMV partly through expression of the constitutively active chemokine receptor US28 induces ß-catenin signaling upon infection. Indeed, mounting evidence links viral infection to ß-catenin hyperactivation. For instance, the Epstein-Barr virus (EBV) activates ß-catenin in latently infected B lymphocytes [39]. The human papillomavirus (HPV) E6 and E7 oncogenes appear to contribute to activation of ß-catenin signalling in HPV16-positive oropharyngeal squamous carcinoma cells [40] and the hepatitis C virus (HCV) encoded core protein potentiates Wnt/ß-catenin signalling in hepatocellular carcinoma cells [41]. For the human immunodeficiency virus (HIV), however, active Wnt/ß-catenin signaling plays a significant role in repression of HIV-1 replication in multiple cell targets [42], [43].

Interestingly, expression of the HCMV-encoded chemokine receptor US28 in intestinal epithelial cells promotes intestinal neoplasia in transgenic mice [26], which is associated with increased accumulation of ß-catenin in the nucleus. In this study we show that this viral receptor leads to activation of ß-catenin and enhanced ß-catenin-dependent transcription in a manner distinct from conventional Wnt-mediated signalling when expressed in NIH3T3 cells or HEK293T cells. Classical Wnt-mediated ß-catenin signaling entails the phosphorylation of LRP 5/6, ultimately leading to the nuclear accumulation of ß-catenin [1], [44]. The absence of LRP6 phosphorylation in US28-expressing cells supports the notion that US28 activates the ß-catenin pathway through alternative routes. Unlike some of the lysophosphatidic acid, prostaglandin and protease activated receptors shown to stabilize ß-catenin at the level of the destruction complex [12], US28-induced TOPflash activation is not PI3K- nor PKC-dependent. COX-2, via concomitant production of prostaglandins and activation of their cognate receptors in US28 expressing cells (Maussang, Langemeijer et al. 2009) is partially involved and does not account for the large increase in ß-catenin activity observed in US28 expressing cells.

In this study it is shown for the fist time that a GPCR, the viral chemokine receptor US28, activates ß-catenin signaling through the Rho-ROCK pathway. Our data show that coupling of US28 to both Gα_q_ and Gα_12/13_ proteins is essential for the observed activation of ß-catenin signaling. Overexpression, scavenging and/or downmodulation of either G protein greatly affect US28 mediated ß-catenin signaling. The reported ligand-independent, constitutive, activity displayed by US28 is primarily exerted through activation of Gα_q_ proteins [30], [45]. Earlier, US28 was shown to also constitutively activate the serum response factor via Gα_q_ proteins and RhoA, the small G protein that is activated by Gα_q_ proteins through RhoGEF [36], [38]. The ligand-dependent activity of US28 directs smooth muscle migration via Gα_12/13_ and RhoA [46]. Interestingly, several regulators of the Wnt/ß-catenin signaling pathway were found to be associated with pro-migratory signaling of US28 via activation of the Pyk2 kinase [47]. In view of the importance of Gα_q_ and Gα_12/13_ in US28 mediated ß-catenin signaling and reported coupling of US28 to RhoA we postulated a role for its downstream target Rho kinase (ROCK) in US28 mediated activation of ß-catenin. Inhibition of ROCK, with the specific inhibitor Y27632, substantiates a role for the Rho-ROCK axis in the US28 induced activation of the ß-catenin pathway. Exposure of US28 expressing cells to the chemokine CCL5 result in further increases in ß-catenin signaling, infering involvement of Gα_12/13_ proteins which is in line with previous studies indicating the involvement of these G proteins in US28-mediated responses [48]. Altogether, our studies demonstrate that US28 activates ß-catenin signaling in a ligand dependent and independent manner, involving Gα_12/13_ and Gα_q_ proteins converging at RhoA/ROCK (**Fig. 5**). Additional experiments are currently ongoing to elucidate the molecular mechanism by which ROCK stabilizes ß-catenin and induces TOPflash activation.

Of importance is that significant increases of ß-catenin stabilization and signaling are observed in HCMV-infected HFFs and glioblastoma cells. This increase in ß-catenin signaling upon HCMV infection is mediated to a large extent by expression of US28, as shown using the deletion mutant HCMV-?US28. Besides US28, other CMV encoded proteins, including another viral GPCR UL33, also contribute to the observed increase in β-catenin signalling upon HCMV infection (**Fig. S3**). Increases in ß-catenin nuclear localization were also reported upon infection of murine CMV [49], reinforcing a role of HCMV-encoded proteins, like US28, in regulating ß-catenin signaling. Sustained activation of the Wnt/ß-catenin pathway induced by gain-of-function mutations of activators of the Wnt pathway, for e.g. mutations in the ß-catenin gene that enhance its stability, or mutations in genes that control ß-catenin stability like APC, the Axins, or E-cadherin, is found in various cancers [3], [4]. Hence, the ability of US28 to constitutively activate ß-catenin signaling, as well as other oncogenic signaling pathways [22], [23], [24], [27], [50] illustrates that this viral receptor may contribute to a malignant phenotype in HCMV positive cells. The fact that expression of US28 promotes development of intestinal dysplasia and cancer in transgenic mice [26] suggests that CMV infection may facilitate development of intestinal neoplasia in humans. Moreover, ß-catenin and components of the Wnt canonical pathway are commonly overexpressed in glioblastoma multiforme [51]. The high incidence of HCMV infection and detection of expression of US28 in human glioblastomas [23], [24] further underscores the relevance of this receptor in cancer development.

Taken together, in this study we have shown an alternative regulation of the ß-catenin pathway. The viral chemokine receptor US28, induces activation of ß-catenin, via the Rho-ROCK pathway. By expression of viral receptor proteins viruses might be able to rewire ß-catenin signaling, contributing to malignant phenotypes.

## Supporting Information

Figure S1 **Wnt3a induces LRP6 phosphorylation at serine 1490 in US28-transfected NIH3T3 cells.** Mock cells and US28 expressing cells were treated with 500 ng/ml recombinant Wnt3a. Subsequently, LRP6 Ser^1490^ phosphorylation was analysed by Western blot analysis.
(EPS)

Figure S2 **Back titration of wild-type HCMV and HCMV ΔUS28.** The levels of infection were assessed by staining for Immediate Early (IEA). The amount of IEA+ cells is not significantly different between the different viral strains. This backtitration was performed on the samples that were used for the analysis shown in Figure 4B.
(EPS)

Figure S3 **Activation of Tcf/Lef by UL33.** UL33 and TOPflash were co-transfected, and luciferase activity was analysed 24 hours post-transfection. UL33 induces Tcf/Lef activation strongly.
(EPS)
